# Atypical cytologic mantle cell lymphoma presentation

**DOI:** 10.1002/jha2.526

**Published:** 2022-07-04

**Authors:** Mathias Chea, Maxime Loyens

**Affiliations:** ^1^ Nimes Caremeau Hospital Montpellier University Montpellier France

1

A 61‐year‐old patient presents with monoclonal B lymphocytosis diagnosed in 2014 with initial moderate splenic involvement without any clinical symptoms.

In 2021, during a follow‐up workup, a Positron Emission Tomography scan is performed objectifying homogeneous splenic involvement with moderate colonic hypermetabolism.

Cytology showed a monoclonal lymphocytosis composed of small lymphocytes with mature chromatin and high nucleocytoplasmic ratio regularly displaying small cytoplasmic expansions (A: May‐Grunwald‐Giemsa stain, 50× objective, B: May‐Grunwald‐Giemsa stain, 100× objective).

Immunophenotyping brought to light a monoclonal population that is CD19^+^ CD20^+^ CD5^+^ CD23^−^ CD43^−^ CD10^−^ CD200^−^ CD38− with a Matutes score at 1/5. Furthermore, cytogenetic analysis was performed showing an *IGH/CCND1* translocation (11;14) with loss of TP53 in all nuclei analyzed. These elements are all in favor of mantle cell lymphoma diagnosis.

However, this cytological presentation is very atypical, the presence of small cells with blebs not being classical in this lymphoma but more in prolymphocytic leukemia for example.

This case thus presents a very particular cytological aspect of mantle cell lymphoma blood dissemination and the importance of cytogenetic analysis and immunophenotyping in addition to cytology (Figure 1).

**FIGURE 1:**
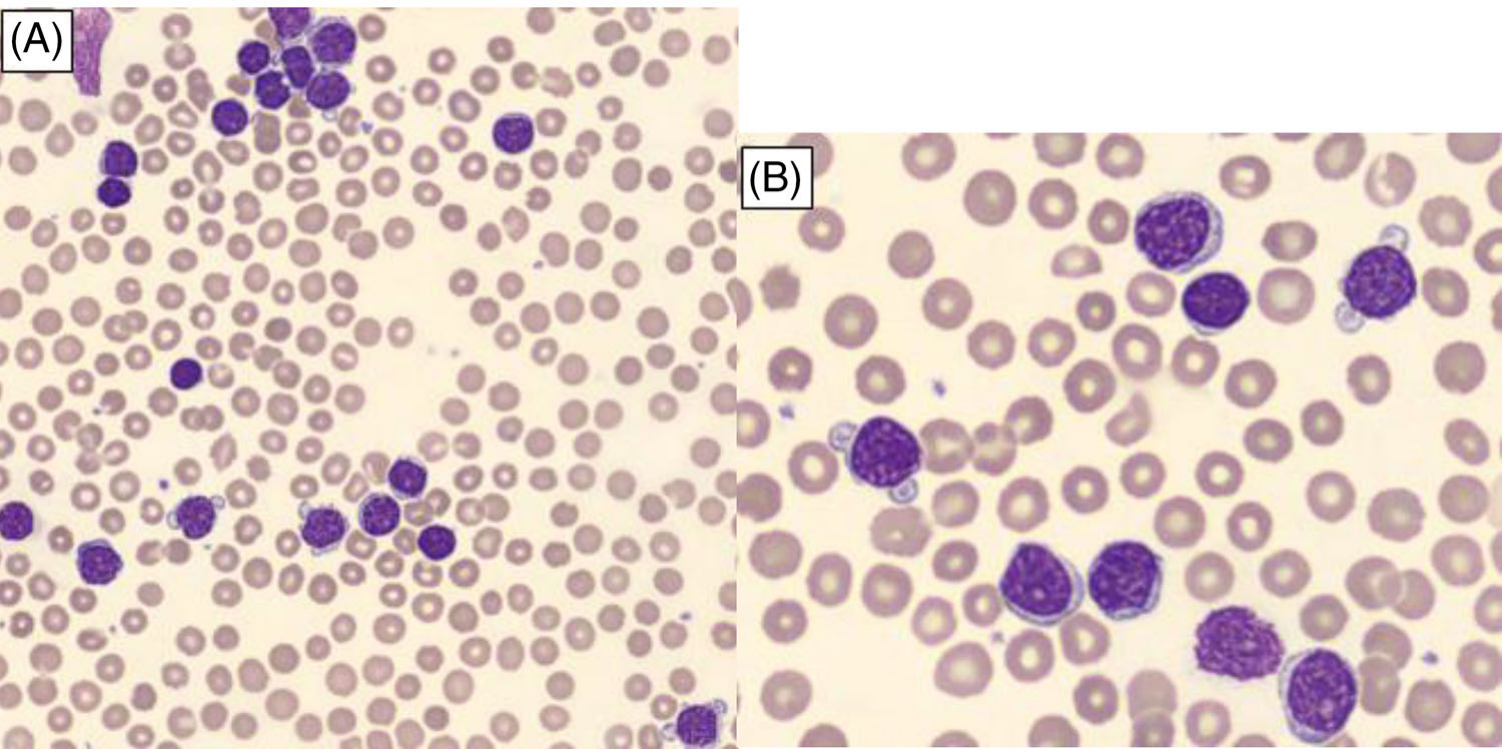
Atypical cells in mantle cell lymphoma (Giemsa stain, panel A: original magnification ×50 and panel B: original magnification ×100)

## CONFLICT OF INTEREST

The data that support the findings of this study are available on request from the corresponding author.

## FUNDING INFORMATION

The authors received no specific funding for this work.

## ETHICS STATEMENT

No research on human was performed on this study.

## AUTHOR CONTRIBUTIONS

Dr Chea Mathias wrote the manuscript and took pictures. Dr Loyens helped with cytometry analysis.

## Data Availability

No new data were created or analyzed in this study. The data that support the findings of this study are available on re‐quest from the corresponding author.

